# Harnessing the power of an X-ray laser for serial crystallography of membrane proteins crystallized in lipidic cubic phase

**DOI:** 10.1107/S2052252520012701

**Published:** 2020-10-15

**Authors:** Ming-Yue Lee, James Geiger, Andrii Ishchenko, Gye Won Han, Anton Barty, Thomas A. White, Cornelius Gati, Alexander Batyuk, Mark S. Hunter, Andrew Aquila, Sébastien Boutet, Uwe Weierstall, Vadim Cherezov, Wei Liu

**Affiliations:** a Center for Applied Structural Discovery at the Biodesign Institute, Arizona State University, Tempe, AZ 85287-1604, USA; b Bridge Institute, Michelson Center for Convergent Bioscience, University of Southern California, 1002 W. Childs Way, Los Angeles, CA 90089, USA; c Center for Free-Electron Laser Science, Deutsches Elektronen-Synchrotron DESY, Notkestraße 85, 22607 Hamburg, Germany; d LCLS, SLAC National Accelerator Laboratory, 2575 Sand Hill Road, Menlo Park, CA 94025, USA; e Department of Chemistry, University of Southern California, Los Angeles, CA 90089, USA; f School of Molecular Sciences, Arizona State University, Tempe, AZ 85287, USA

**Keywords:** G-protein-coupled receptors, membrane proteins, XFELs, serial femtosecond crystallography, adenosine A_2A_ receptors, lipidic cubic phases, high dynamic range detectors

## Abstract

Serial femtosecond crystallography (SFX) with X-ray free-electron lasers (XFELs) has proven highly successful for structure determination of challenging membrane proteins crystallized in lipidic cubic phase; however, like most techniques, it has limitations. Here we attempt to address some of these limitations related to the use of a vacuum chamber and the need for attenuation of the XFEL beam, in order to further improve the efficiency of this method. Using an optimized SFX experimental setup in a helium atmosphere, the room-temperature structure of the adenosine A_2A_ receptor (A_2A_AR) at 2.0 Å resolution is determined and compared with previous A_2A_AR structures determined in vacuum and/or at cryogenic temperatures. Specifically, the capability of utilizing high XFEL beam transmissions is demonstrated, in conjunction with a high dynamic range detector, to collect high-resolution SFX data while reducing crystalline material consumption and shortening the collection time required for a complete dataset. The experimental setup presented herein can be applied to future SFX applications for protein nanocrystal samples to aid in structure-based discovery efforts of therapeutic targets that are difficult to crystallize.

## Abstract 2

Serial femtosecond crystallography of adenosine A_2A_ receptor (A_2A_AR) crystallized in lipidic cubic phase was performed using high X-ray free-electron laser transmission with a high dynamic range detector in a helium atmosphere. The 2.0 Å resolution A_2A_AR structure model is presented and compared with previous A_2A_AR structures determined in a vacuum and/or at cryogenic temperatures.

## 1. Introduction   

Elucidating high-resolution X-ray structures of G-protein-coupled receptors (GPCRs) and other membrane proteins using synchrotron radiation sources has been limited by the difficulty of obtaining high-quality crystals that can withstand radiation damage. So far, only a few GPCR structures have been resolved to better than 2.0 Å resolution using synchrotron radiation (Liu *et al.*, 2012 ▸; Fenalti *et al.*, 2014 ▸; Segala *et al.*, 2016 ▸; Rucktooa *et al.*, 2018 ▸; Weinert *et al.*, 2017 ▸). Several challenges must be overcome during crystallization and diffraction data collection to achieve high-resolution structure models. First, the size of a protein crystal suitable to resolve a 3.5 Å structural model using synchrotron diffraction should be at least 20 µm in each dimension (Sliz *et al.*, 2003 ▸). Additionally, as secondary radiation damage propagates throughout the crystals, diffraction data quality deteriorates, resulting in decreased resolution, and increased unit-cell volume, *B* factors and mosaicity (Garman & Owen, 2006 ▸). Typically, protein crystals are cryo-cooled to reduce secondary radiation damage during data collection. However, subjecting protein crystals to cryogenic conditions can potentially introduce non-physiological artefacts, owing to improper freezing, and increase their mosaicity (Watenpaugh, 1991 ▸).

Recent advances at synchrotron microfocus beamlines have allowed room-temperature serial diffraction data collection using crystals ∼10 µm in size (Yamamoto *et al.*, 2017 ▸; Miller *et al.*, 2019 ▸). Serial millisecond crystallography (SMX) tech­niques have enabled room-temperature structure determination of GPCRs at moderate resolutions using synchrotron radiation sources (Martin-Garcia *et al.*, 2017 ▸; Weinert *et al.*, 2017 ▸). Nonetheless, the crystal sizes needed for collecting high-resolution SMX data are much larger than those required for serial femtosecond crystallography (SFX) (Martin-Garcia *et al.*, 2017 ▸; Weinert *et al.*, 2017 ▸). An X-ray free-electron laser (XFEL) source with extremely bright femtosecond pulses allows for diffraction patterns to be collected from protein crystals with minimal deleterious effects, as outlined above, specifically with the intent of minimizing radiation damage, termed ‘diffraction before destruction’ (Weierstall *et al.*, 2014 ▸; Neutze & Hajdu, 1997 ▸). Also, SFX experiments are typically conducted at room temperature, permitting a more native-like temperature environment for the protein target of interest. Over the last several years, SFX has demonstrated a clear advantage for structure determination of difficult for crystallization membrane proteins such as GPCRs (Stauch & Cherezov, 2018 ▸).

Despite the numerous advantages offered by SFX, further technical advancements are critical to optimize data collection. One major limitation observed in SFX experiments at the Linac Coherent Light Source (LCLS) using the Cornell–SLAC pixel array detector (CSPAD) (Carini *et al.*, 2014 ▸; Blaj *et al.*, 2015 ▸) is the need to attenuate the beam to ∼10% (a few hundred µJ pulse^−1^ at the sample) of its full power (Martin-Garcia *et al.*, 2016 ▸; Stauch & Cherezov, 2018 ▸; Coe & Ros, 2018 ▸). This attenuation is required to avoid detector pixel saturation and possible damage by the strong low-resolution diffraction spots and to reduce the lipidic cubic phase (LCP) flow disruption caused by the highly intense XFEL beam (Stan *et al.*, 2016 ▸; Stauch & Cherezov, 2018 ▸). Specifically, owing to the viscous nature of the LCP matrix, interaction with a strong XFEL beam can lead to disruption of the LCP stream, sticking it to the injector nozzle, which requires stopping the experiment to clean the nozzle, thereby increasing the data-collection time and negating any advantage in sample consumption that the method offers. Furthermore, beam attenuation is undesirable when attempting to collect high-resolution data (<2.0 Å), as the weaker high-resolution diffraction spots become harder to detect (Fromme, 2015 ▸) since each spot is recorded at a lower signal-to-background ratio. Since diffraction intensity typically scales with crystal size, attenuating beam fluence for SFX experiments further limits sample crystal sizes that can yield quality diffraction patterns as the signal-to-noise ratio decreases (Coe & Ros, 2018 ▸).

Lastly, since the available beam time at XFEL sources is scarce, it is critical to increase their usage efficiency. Therefore, in this experiment we tested a secondary chamber with a helium atmosphere environment at the LCLS coherent X-ray imaging (CXI) instrument (Liang *et al.*, 2015 ▸), in which the 1 µm focused XFEL beam that passed through the sample in the main vacuum sample chamber is re-focused by beryllium lens to a spot of <3 µm. While not carried out in our experiment, the refocused beam in the secondary helium chamber can be used for simultaneous data collection with the focused beam in the primary vacuum chamber, thus doubling the diffraction sample throughput during available XFEL beam time (Boutet *et al.*, 2015 ▸; Hunter *et al.*, 2016 ▸). Additionally, the utilization of a high dynamic range Rayonix MX170-HS detector allowed for diffraction data to be collected using an unattenuated XFEL beam, although the beryllium lens and the diamond window allowing for the passage through the upstream chamber contributed to an overall beam attenuation by a factor of two. In this study, we present the 2.0 Å model of the human adenosine A_2A_ receptor (A_2A_AR) using SFX data collected in a helium environment under atmospheric pressure and at room temperature. We compare this model with the 1.8 Å synchrotron structure [PDB entry 4eiy (Liu *et al.*, 2012 ▸)] as well as with other published A_2A_AR structures from XFEL SFX [PDB entries 5nm4 (Weinert *et al.*, 2017 ▸) and 5k2d (Batyuk *et al.*, 2016 ▸)] and synchrotron SMX experiments [PDB entry 5nlx (Weinert *et al.*, 2017 ▸)].

## 2. Results   

### 2.1. Crystal sample generation and SFX experimental setup   

**Figure 1:**
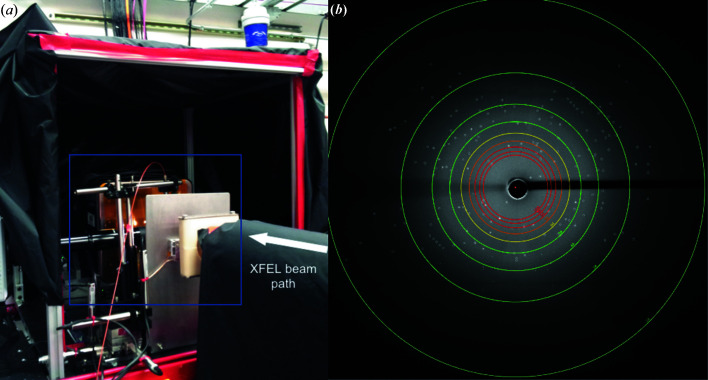
(*a*) A view of the experimental setup for LCP-SFX data collection in helium at CXI. A detailed view of the instrumentation in the blue inset from (*a*) is shown in Fig. S1. (*b*) A representative diffraction pattern during data collection. Diffraction spots located by *Cheetah* are circled.

Microcrystal samples of human A_2A_AR in complex with the antagonist ZM241385 for SFX experiments were generated using the same methodology and crystallization conditions as previously reported (Liu *et al.*, 2012 ▸, 2014 ▸). At the LCLS CXI instrument, microcrystals measuring ∼5 × 5 × 2 µm were combined to produce 40 µl of densely packed LCP-crystal sample. The samples were loaded and injected into the XFEL beam using an LCP injector as previously described (Weierstall *et al.*, 2014 ▸), with the major exception being that the injector was housed in a helium-filled enclosure [Fig. 1 ▸(*a*), details are shown in Fig. S1 in the Supporting information] instead of the commonly used conventional vacuum chamber for SFX experiments at CXI. A Rayonix MX170-HS detector was used to collect the SFX data at a 2 × 2 binning mode with a data-acquisition rate of 10 Hz. A representative diffraction image at ≃1 mJ recorded to 2.0 Å at the edge of the detector is shown in Fig. 1 ▸(*b*).

### 2.2. Diffraction data collection and processing   

**Figure 2:**
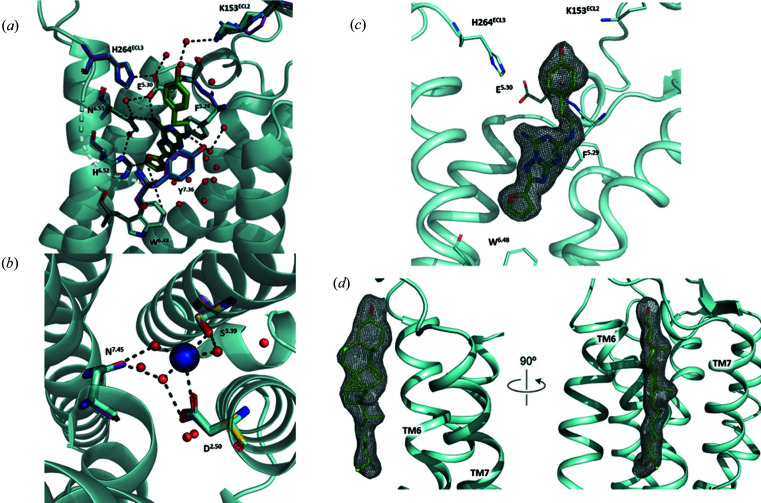
Quality and validation of the A_2A_AR model obtained in this study. (*a*) The ZM341385 binding site is conserved across all models. The ligand from our structure (green sticks) is shown with side chains from all structures aligned. (*b*) Conservation of the sodium binding site. The side chains from all the models are shown with the sodium (blue sphere) and waters (red spheres) from our current model. (*c*) 2*mF*_o_ − *DF*_c_ map contoured at 1.0σ shows the density for ZM341385 in our model. (*d*) 2*mF*_o_ − *DF*_c_ map contoured at 1.0 σ shows clear cholesterol densities in our model.

**Figure 3:**
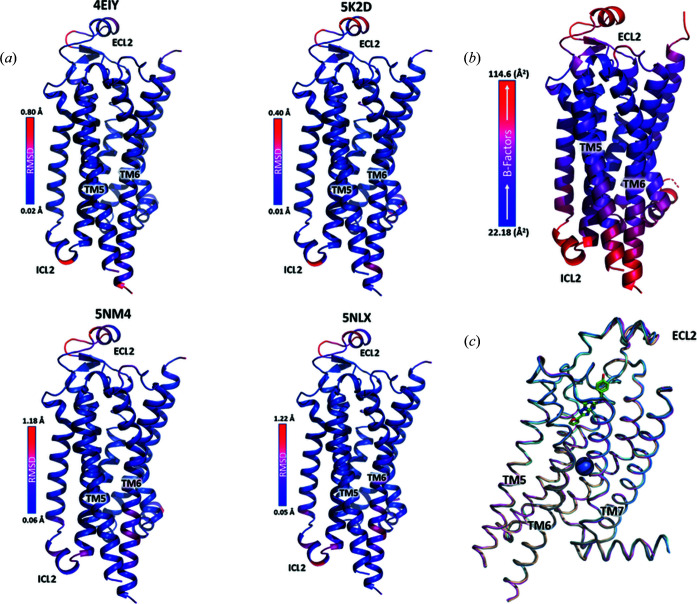
(*a*) Cα RMSD values between the current A_2A_AR structure model and the compared models. Lower RMSD values are shown in blue and higher RMSD values are shown in red. Scale bars are included for maximum and minimum values. (*b*) The current A_2A_AR structure model coloured by *B* factors. Lower *B* factors are shown in blue and higher *B* factors are shown in red. The scale bar shows the minimum and maximum *B* factors. (*c*) Overlay of all A_2A_AR models compared in this study. Our model (cyan), 5nm4 (tan), 5nlx (purple), 4eiy (grey) and 5k2d (blue). The BRIL fusion protein is removed.

From ∼2 h of data collection, we had an average crystal hit rate of 37.5% resulting in 26 341 ‘hits’ – defined as crystal diffraction patterns containing at least 15 peaks with the signal-to-noise ratio above 6. From 26 341 hits, 16 737 patterns were successfully indexed (63.5% indexing rate) and used to build the model presented here. After molecular replacement (MR) and refinement, the electron-density maps revealed three clear densities, corresponding to cholesterol molecules near the receptor, and a density for the ligand ZM241385 consistent with previous structures (Fig. 2 ▸). Densities for lipid molecules, co-purified with the receptor or utilized in crystallization and sample delivery, as well as other molecules (polyethyl­ene glycol and glycerol), were resolved as well. We also observed a sodium ion coordinated by three water molecules and residues Asp52^2.50^ and Ser91^3.39^ [the superscripts refer to the generic Ballesteros–Weinstein numbering scheme for class A GPCRs (Ballesteros & Weinstein, 1995 ▸)] in the conserved allosteric site known to be important for receptor activation (Liu *et al.*, 2012 ▸; Katritch *et al.*, 2014 ▸) (Fig. 2 ▸). For a comprehensive comparison of our model with other existing models in the PDB, we searched for all available A_2A_AR structures bound to ZM241385 and containing the apocytochrome b_562_RIL (BRIL) fusion protein at intracellular loop 3 (ICL3). We then separated these structures according to resolution and diffraction technique. For the sake of brevity, structures with lower than 2.2 Å resolution were not analyzed in detail and are thus excluded from the present discussion. Table 1 ▸ compares the statistics for our model with four other previously published structures: 4eiy, a 1.8 Å structure from merging multiple single-crystal synchrotron diffractions at cryo-conditions (Liu *et al.*, 2012 ▸); 5nm4, a 1.7 Å structure obtained using SFX from an XFEL source (Weinert *et al.*, 2017 ▸); 5k2d, a 1.9 Å SFX structure with crystals delivered in vacuum (Batyuk *et al.*, 2016 ▸); and 5nlx, a 2.14 Å synchrotron SMX structure (Weinert *et al.*, 2017 ▸). Superimposition of our model with these high-resolution structures showed close alignment with low root-mean-square deviation (RMSD) values for C_α_ atoms (RMSD values for all atoms are shown in parentheses): 0.281 (0.683) Å, 0.279 (0.651) Å, 0.193 (0.814) Å and 0.082 (0.571) Å, between the current structure and 5nm4, 5nlx, 4eiy and 5k2d, respectively [Fig. 3 ▸(*a*)]. Overall, all the models were found to be in agreement with each other without any significant observable differences between the synchrotron single-crystal diffraction method, SMX and SFX structures. We observed similar crystallographic statistics between the models, with higher *B* factors for structures determined at room temperature compared with cryogenic conditions (4eiy), as expected (Table 1 ▸). Our final model was refined to 2.0 Å with similar crystallographic statistics as the other A_2A_AR models (Table 1 ▸).

## 3. Discussion   

After refinement, we observed no significant differences in the 2*mF*_o_ − *DF*_c_ maps between our model and previously published A_2A_AR structures (4eiy, 5k2d, 5nlx and 5nm4). Analysis of structural characteristics such as di­sulfide bonds, the sodium binding pocket, ligand binding residues and cholesterol molecules showed similar quality between the 2*mF*_o_ − *DF*_c_ maps (Fig. S2). We observed slightly larger RMSD values in regions including ICL2, the intracellular portion of TM6 and ECL2 [Fig. 3 ▸(*a*)]. Moreover, weaker densities were observed across all the models for ICL2 and ECL2, potentially indicative of the dynamic nature of these loops. These regions of weaker density and higher RMSD also correlate to higher *B* factors from the model [Fig. 3 ▸(*b*)]. Nonetheless, all the structure models generated using different diffraction methods are comparable, with important structural characteristics clearly resolved (Fig. 2 ▸). Fig. 3 ▸(*c*) shows a ribbon representation of all the structure models aligned to our current model.

At the time of writing, the standard detector installed in the primary chamber of the LCLS CXI instrument is the CSPAD, capable of high-speed readout at 120 Hz (Liang *et al.*, 2015 ▸; Blaj *et al.*, 2013 ▸). The main advantage of this detector is that it is specifically made for LCLS applications; it has a large cross-sectional area (1516 × 1516 pixels at 110 µm pixel^−1^; 167 × 167 mm), can count single photons, with a maximum signal of 2700 photons (8 keV) pixel^−1^, and has a fast 120 Hz data-acquisition rate, all of which are amenable for XFEL SFX experiments. The results reported here were recorded using the Rayonix MX170-HS detector. Beyond the technical differences (CCD versus pixel arrays), which are outside the scope of this study, we mainly focus on the advantages the MX170-HS detector offers. During our SFX experiments, the MX170-HS detector was recording in a 2 × 2 binning mode (1920 × 1920 pixels at 89 µm pixel^−1^; 171 × 171 mm) which has a capacity for recording a max signal of 50 000 photons (12 keV) pixel^−1^ (Blaj *et al.*, 2013 ▸); in comparison, the CSPAD can record either a max signal of 2700 photons (8 keV) pixel^−1^ at the low-gain mode or 350 photons (8 keV) pixel^−1^ at the high-gain mode, significantly less than that of the MX170-HS detector. Additionally, the lower dynamic range of the CSPAD limits the amount of tolerable background noise since a detector readout must include a full LCLS pulse, an elevated background can utilize all of the dynamic range of the detector and lead to signals above background saturating the detector. Performing experiments in vacuum, as regularly carried out at XFELs, alleviates this problem. In contrast, our experiment was performed at ambient pressure in a helium atmosphere. Utilizing the high dynamic range of the MX170-HS detector allowed us to overcome the background scattering effects contributed by helium atoms.

Using the MX170-HS detector coupled with a full strength XFEL beam, we were able to resolve an A_2A_AR structure model to 2.0 Å with reasonable statistics, demonstrating the capabilities of the hardware setup presented herein. The A_2A_AR crystal sample used in this study is comprised of microcrystals that average ∼5 × 5 × 2 µm in size. In contrast, the crystals used to generate the 5nm4 model from SFX were reported to be 30 × 30 × 5 µm (Weinert *et al.*, 2017 ▸). Optimizing conditions to grow larger crystals is often a time-consuming process that may take months to years and is a significant bottleneck in protein structural studies. Our present method alongside established SFX methods have shown the potential in obtaining high-resolution diffraction data by focusing on optimizing crystal growth conditions to form dense uniform showers of small crystals. Data collection under the conditions outlined here has numerous benefits. First, the high dynamic range detectors can record the intense diffraction signals at low resolution so the images can be collected using an unattenuated beam without concerns for damaging the detector electronics. Second, although not tested explicitly here, when more diffraction spots are observed in each image, fewer images should be required for structure determination, which could reduce the sample consumption and the data-collection time. It has been common to collect >10^4^ SFX diffraction images to enable the building of a quality structure model, although protein structures have been resolved from SFX using <10^4^ diffraction images on occasion (Coe & Ros, 2018 ▸). Although the MX170-HS detector had a slower frame rate (10 Hz), its higher dynamic range can make the MX170-HS detector a better choice for SFX experiments seeking to record high-quality resolution data using a higher flux of the FEL beam at atmospheric pressure. The previously published 1.9 Å A_2A_AR SFX model (PDB entry 5k2d) (Batyuk *et al.*, 2016 ▸) was built using diffraction data from similarly sized crystals, formed in similar crystallization conditions as the present study, collected for ∼2 h using a 9.8 keV FEL beam and the CSPAD. Similarly, our model was built at 2.0 Å resolution using SFX data from ∼2 h of beam time (Table 1 ▸). Despite the differences between the detectors and data-acquisition rates used to record SFX data, our model and 5k2d are in high agreement, as previously discussed. If we extrapolate our results with the MX170-HS detector to a 120 Hz repetition rate, it may be possible to collect a similar dataset in only 10 min of beam time. This is suggestive of the potential of this methodology to deliver high-resolution membrane protein crystal structures while minimizing SFX data-collection time and sample consumption. Generally, the ideal detector for SFX data collection should have a high dynamic range, low read noise and a high acquisition rate matching or exceeding the full pulse rate of the XFEL source. The newest generation of XFEL beamlines (LCLS-II/SHINE) are capable of repetition rates at or above 100 kHz. For new detectors to be able to match the repetition rates of these new machines, the physical dimensions of the detectors and the total amount of pixels could be diminished while increasing the pixel size (Bergamaschi *et al.*, 2020 ▸). Theoretically, a 1 megapixel array (1000 × 1000 pixels) could still achieve a 100 kHz repetition rate (Bergamaschi *et al.*, 2020 ▸). Specifically, the JUNGFRAU 4M detector (Leonarski *et al.*, 2018 ▸) is an example of a detector that combines a high dynamic range with a higher repetition rate (120 Hz), and may allow future users to collect high-resolution diffraction data from crystals that were previously shown to provide weak low-resolution diffraction at highly attenuated XFELs. The JUNGFRAU 4M detector will become the default detector for sample chamber 1 (SC1) at the CXI instrument in mid-2020.

An additional benefit to our method is the better measurement of low-resolution diffraction spots from better intensity estimates owing to the aforementioned detector characteristics. We observed that the indexing rate from our experiment was 63.5% while the other A_2A_AR models generated from SFX, 5k2d and 5nm4, showed indexing rates of 31.3% and 2.3%, respectively (Weinert *et al.*, 2017 ▸) (Table 1 ▸). Despite the higher indexing rate, our overall 〈*I*/σ(*I*)〉 is lower than that of 5k2d (Table 1 ▸), possibly because of background scattering from the helium path between the crystal and the beam stop (Perutz & Rogers, 1946 ▸). Furthermore, it has been shown that acquiring more images can improve the signal-to-noise ratio of the dataset (Glynn & Rodriguez, 2019 ▸). In our dataset we used fewer images (16 737) compared with 5k2d (72 753).

A central problem around SFX is the scarcity of XFEL facilities and the difficulty in obtaining experimental beam time. Enabling the use of the full power of the XFEL source, as demonstrated in this work, along with a high dynamic range and high repetition rate detector should greatly increase the efficiency of SFX experiments for membrane protein microcrystals grown and delivered in LCP matrix. Additionally, simultaneous data collection in the primary vacuum sample chamber at CXI as well as in the secondary helium-filled chamber using the refocused beam should further increase the number of experiments conducted at the facility. The methods and hardware setup presented here have led to the development of the macromolecular femtosecond crystallography (MFX) instrument (Sierra *et al.*, 2019 ▸) at LCLS as well as the secondary serial sample chamber (Boutet *et al.*, 2015 ▸; Liang *et al.*, 2015 ▸) at CXI to reuse the XFEL beam. Lastly, XFEL-SFX experiments have been regularly conducted in a helium atmos­phere at SACLA, further demonstrating the utility of performing XFEL-SFX experiments in a helium environment (Tono *et al.*, 2015 ▸; Sugahara *et al.*, 2017 ▸; Shimazu *et al.*, 2019 ▸).

## 4. Methods   

### 4.1. Adenosine A_2A_-BRIL receptor purification and crystallization   

Receptor purification and crystallization followed previously published protocols (Liu *et al.*, 2012 ▸). Briefly, 1 l scale insect cell membranes were prepared as described, and solubilized in a buffer containing 50 m*M* HEPES at pH 7.5, 800 m*M* NaCl, 2 m*M* theophylline (Sigma), 1.0 mg ml^−1^ iodo­acetamide (Sigma), EDTA-free cOmplete protease inhibitor cocktail (Roche), 1%(*w*/*v*) *n*-do­decyl-β-d-malto­pyran­o­side (DDM, Anatrace) and 0.2%(*w*/*v*) cholesteryl hemisuccinate (CHS, Sigma) for 3 h at 4°C. The insoluble material was removed by centrifugation at 250 000*g* for 45 min and the supernatant was incubated with TALON affinity chromatography resin (Takara–Clontech) overnight in the presence of 20 m*M* imidazole. The resin was washed using successive volumes of buffers containing 100 µ*M* of ZM241385 (Tocris, prepared as 100 m*M* stock in di­methyl sulfoxide) with increasing concentrations of imidazole. A_2A_AR was eluted in the elution buffer [50 m*M* HEPES pH 7.5, 800 m*M* NaCl, 10% glycerol, 100 µ*M* of ZM241385, 0.01%(*w*/*v*) DDM, 0.002%(*w*/*v*) CHS, 300 m*M* imidazole] and subsequently concentrated to ∼40 mg ml^−1^ using an Amicon centrifugation concentrator (100 kDa molecular weight cutoff; MilliPore).

The receptor was reconstituted in LCP by mixing with a lipid mixture consisting of 90%(*w*/*w*) monoolein and 10%(*w*/*w*) cholesterol at a ratio of 2 parts protein to 3 parts lipid by volume using a lipid syringe mixer (Caffrey & Cherezov, 2009 ▸). The sample was then subjected to crystallization in gas-tight Hamilton syringes, as previously described (Liu *et al.*, 2014 ▸; Batyuk *et al.*, 2016 ▸). Each crystallization syringe contained ∼5 µl of the LCP sample with 50 µl of the following precipitant solutions: 0.1*M* sodium citrate pH 5.0, 26% or 28% PEG400, and either 30 m*M*, 50 m*M* or 60 m*M* sodium thio­cyanate. All syringes were sealed and incubated at 20°C with crystal formation observed within 24 h. All crystal samples were consumed within the allotted experiment time at LCLS.

### 4.2. XFEL-SFX diffraction data collection   

Gas-tight Hamilton syringes containing A_2A_AR microcrystals grown in LCP were transported to LCLS at 20°C inside a ThermoSafe Greenbox (Sonoco). After removing precipitant solutions, samples from 3–4 syringes were combined together and titrated with a few microlitres of monoolein to absorb the residual precipitant and ensure that the sample remains in cubic phase. The final sample was loaded in the reservoir of an LCP injector (Weierstall *et al.*, 2014 ▸), which was mounted in a helium enclosure at the CXI instrument. The unattenuated XFEL beam was refocused with four compound refractive lenses of 50 µm radius of curvature (Chapman *et al.*, 2011 ▸) for a total focal length of 1.79 m. These lenses were placed 3 m downstream of CXI’s 1 µm focus and 4.42 m upstream of the sample. The beam size on the sample was estimated to be just below 3 µm owing to the lens chromatic aberration and assuming a 30 eV bandwidth. The nominal FEL beam pulse energy exiting the undulator was ∼2 mJ and estimated to be ∼1 mJ at the focus. The SFX diffraction data were collected using a high dynamic range detector (Rayonix MH170-HS) at 10 Hz with a sample flow rate of 0.2 µl min^−1^, under a helium path and normal atmos­pheric pressure.

### 4.3. Data processing and model building   

The SFX data were first processed with *Cheetah* (Barty *et al.*, 2014 ▸) to delineate patterns containing crystal diffraction, termed ‘hits’, from the rest of the patterns using the following settings: *peakfinder8*, a threshold of 50 detector units of intensity, a minimum signal-to-noise ratio of 6, minimum number of peaks of 15, minimum pixels peak^−1^ of 2 and a local background radius of 4. 26 341 hits were found, with an average hit rate of 37.5%. *CrystFEL* (version 0.8.0+049c3eb4) was used for indexing and integration (integration radii of 4, 5 and 7) based on the peaks found by *Cheetah* (White, 2019 ▸; White *et al.*, 2016 ▸). 16 737 patterns were successfully indexed using a combination of *MOSFLM* (Powell *et al.*, 2013 ▸), *DirAx* (Duisenberg, 1992 ▸), *XDS* (Kabsch, 2010 ▸), *asdf* (White *et al.*, 2016 ▸) and *XGANDALF* (Gevorkov *et al.*, 2019 ▸). The sample-to-detector distance along with detector geometry were optimized using *geoptimiser* (Yefanov *et al.*, 2015 ▸) with lysozyme crystal diffraction patterns collected at the beginning of the experiment to generate a virtual powder pattern. Multiple indexing runs were performed using finer detector geometry corrections for each indexing run to arrive at the final stream of data. Reflections were scaled and merged using *partialator *with the ‘unity’ model (*i.e.* no partiality modelling), a saturation cutoff of 10 000 detector intensity units and one scaling/merging iteration. Using data up to a resolution of 2.0 Å, an initial model was generated by MR phasing using the 1.9 Å XFEL structure (PDB entry 5k2d) modified to a polyalanine model, as the search model in the *Phaser* MR module [*Phenix* version 1.17 (McCoy *et al.*, 2007 ▸)] in order to reduce phase bias (Adams *et al.*, 2010 ▸, 2011 ▸). Iterative cycles of model refinement were carried out using *Phenix.refine* with TLS (translation, libration, screw) refinement parameters in five TLS groups. Manual inspection and model modifications in *Coot* (Emsley & Cowtan, 2004 ▸; Emsley *et al.*, 2010 ▸) were subsequently performed. The ligand, lipid and cholesterol molecules were manually modelled into electron densities also using *Coot*. Data-collection and refinement statistics are presented in Table 1 ▸. The protein structure images presented in the figures were generated using *PyMol* (Schrödinger, 2015 ▸).

## Supplementary Material

Supporting information. DOI: 10.1107/S2052252520012701/it5021sup1.pdf

## Figures and Tables

**Table 1:** Data-collection and refinement statistics for the A_2A_AR models discussed in the present study

	Current model	4eiy	5nm4	5nlx	5k2d
Data collection					
Method/source	SFX/XFEL	Small wedge/synchrotron	SFX/XFEL	SMX/synchrotron	SFX/XFEL
Resolution range (Å)	27.7–2.0 (2.07–2.00)	27.5–1.8 (1.86–1.80)	19.6–1.7 (1.76–1.70)	34.5–2.1 (2.22–2.14)	24.0–1.9 (2.00–1.90)
Space group	*C*222_1_	*C*222_1_	*C*222_1_	*C*222_1_	*C*222_1_
Cell dimensions (Å)					
*a*	40.4	39.4	39.9	40.3	40.4
*b*	180.5	179.5	179.2	180.1	180.7
*c*	142.7	140.3	141.2	142.7	142.8
Crystal size (µm^3^)	5 × 5 × 2	60 × 10 × 3	30 × 30 × 5	30 × 30 × 5	5 × 5 × 2
Unique reflections	35870 (3486)	44252 (4100)	56793 (3437)	32392 (2761)	41882 (2933)
〈*I*/σ(*I*)〉	4.1 (1.22)	17.7 (1.8)	2.93 (0.44)	13.17 (0.7)	6.0 (0.6)
Redundancy	205 (72)	4.0 (3.3)	23.3 (3.0)	1,007 (8.1)	291 (62)
*R*_split_ (%) or *R*_merge_ (%) (4eiy)	19.1 (211)	10 (81)	17.9 (315)	4.7 (212)	10.1 (197)
CC*	0.99 (0.50)	N/A	0.99 (0.45)	0.99 (0.47)	0.99 (0.58)
Completeness (%)	100.0 (100.0)	95.1 (92.8)	94.6 (61.8)	99.5 (95.7)	100.0 (100.0)
Wilson *B* factor (Å^2^)	33.3	23.7	40.4	45.2	41.5
Average *B* factors (Å^2^)					
Overall	48.0	34.0	62.8	69.9	58.5
A_2A_AR	39.4	25.2	50.0	55.8	45.3
BRIL	82.6	55.9	90.4	117.0	92.3
Lipids	72.6	45.4	140.6	98.6	80.1
Ligand	32.9	20.4	37.6	40.5	35.6
Solvent	48.3	37.4	52.8	46.7	54.1
Number of indexed images used for dataset	16737	–	3563	128086	72753
Indexing rate (%)	63.5	–	2.3	10.8	31.3
Number of reflections used in refinement	35862	42032	53302	27734	39840
Reflections used for *R*_free_	2000	2221	2606	1430	1988
*R*_work_/*R*_free_ (%)	19.3/21.6	17.4/21.3	21.2/23.5	19.9/22.9	17.4/20.7
Number of non-hydrogen atoms					
Macromolecules	2978	3105	2899	2894	3121
Lipids	392	456	174	138	397
Ligand	25	25	25	25	25
Solvent	85	185	59	33	93
Protein residues	391	390	382	382	391
RMS bonds (Å), angles (°)	0.010, 1.06	0.016, 1.29	0.014, 1.35	0.014, 1.41	0.010, 1.20
Ramachandran plot analysis					
Favoured (%)	98.2	99.0	98.2	96.6	99.0
Allowed (%)	1.8	1.0	1.6	3.4	1.0
Outliers (%)	0.0	0.0	0.3	0.0	0.0
Rotamer outliers (%)	4.01	0.31	1.37	3.08	2.20
Clashscore	1.30	2.69	3.33	4.51	3.20
