# Supplementary material for: Harnessing the power of an X-ray laser for serial crystallography of membrane proteins crystallized in lipidic cubic phase
Source: IUCrJ. 2020 Oct 15;7(Pt 6):976–84. doi: 10.1107/S2052252520012701 (PMC7642783; doi:10.1107/S2052252520012701)
Supplement: Supplementary file 1 [file m-07-00976-sup1.pdf]

IUCrJ

Volume 7 (2020)

Supporting information for article:

Harnessing the power of an X-ray laser for serial crystallography of membrane proteins crystallized in lipidic cubic phase

Ming-Yue Lee, James Geiger, Andrii Ishchenko, Gye Won Han, Anton Barty, Thomas A. White, Cornelius Gati, Alexander Batyuk, Mark S. Hunter, Andrew Aquila, Sébastien Boutet, Uwe Weierstall, Vadim Cherezov and Wei Liu

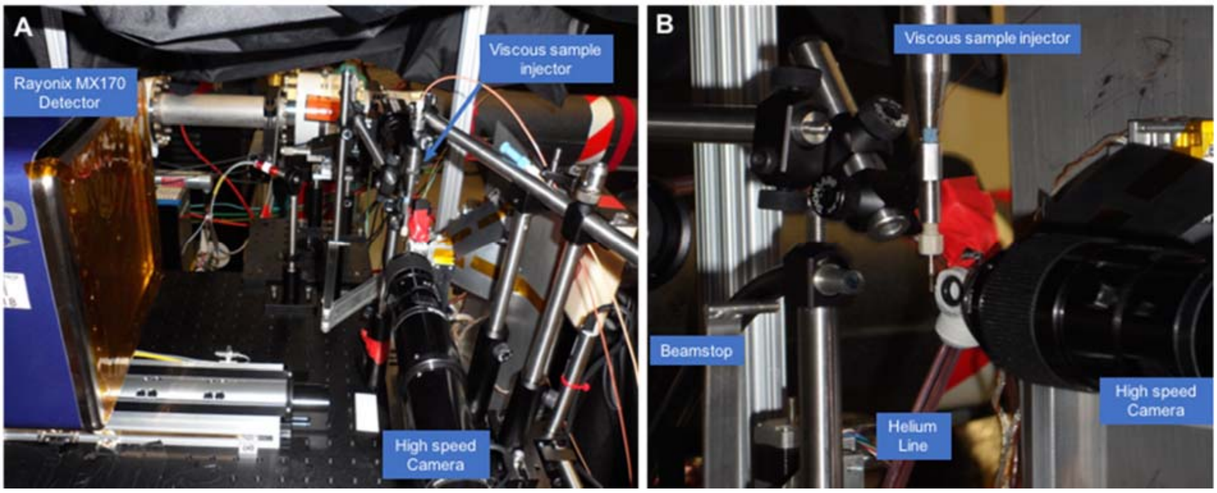

Figure S1 Detailed instrument setup from Figure 1A inset region. A) Side profile view highlighting the location of the detector, viscous sample injector, and other instruments within the sample chamber. B) Another view of the viscous sample injector, beam stop, and helium line inside the sample chamber.

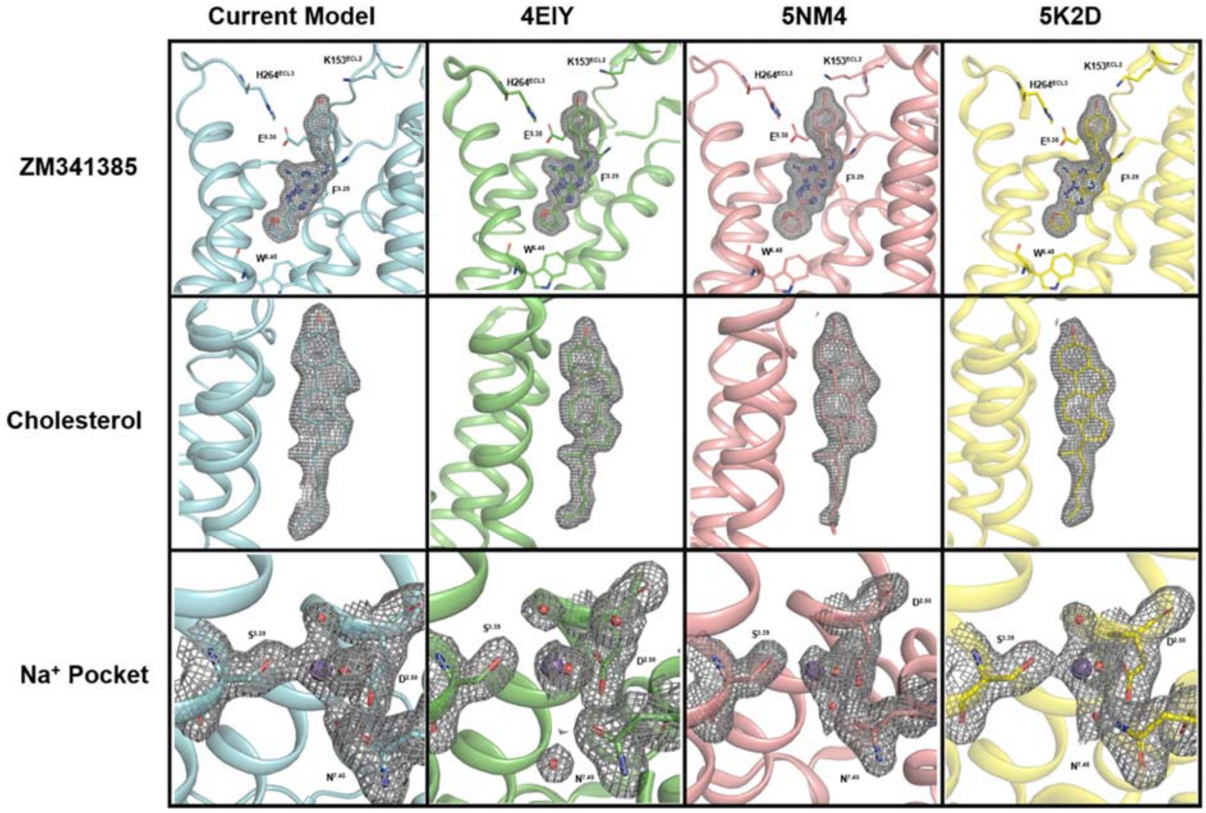

Figure S2 Comparison of the ligand, cholesterol, and sodium pocket $2mF_o$-DF_c electron densities.
